# TOMM40‐G and APOE4 modulate Alzheimer's plasma biomarker levels

**DOI:** 10.1002/alz70856_099618

**Published:** 2025-12-25

**Authors:** Marcella A. Barneclo, Nancy E Ortega, Iris J. Broce‐Diaz, Judy Pa

**Affiliations:** ^1^ Alzheimer's Disease Cooperative Study, La Jolla, CA, USA; ^2^ Alzheimer's Disease Cooperative Study (ADCS), University of California San Diego, La Jolla, CA, USA; ^3^ Neurosciences Graduate Program, University of California San Diego, La Jolla, CA, USA; ^4^ University of California, San Diego, La Jolla, CA, USA; ^5^ Alzheimer's Disease Cooperative Study (ADCS), University of California, San Diego, La Jolla, CA, USA; ^6^ Neuroscience Graduate Program, University of California San Diego, La Jolla, CA, USA

## Abstract

**Background:**

The *TOMM40* gene has been linked to Alzheimer's disease (AD), with the G allele of SNP rs2075650 identified as a risk variant. Accordingly, *TOMM40*‐G carriers exhibit an increased risk of AD, potentially through mitochondrial dysfunction contributing to neurodegeneration and age‐related diseases. However, the association between *TOMM40*‐G and plasma biomarkers of AD remains limited, and the moderating effect of sex and APOE4 on these associations is relatively unknown.

**Method:**

The study included 1,149 cognitively unimpaired older adults from the Anti‐Amyloid Treatment in Asymptomatic Alzheimer's Disease (A4) cohort (39.8% women; mean age=71.5 ± 4.69 years, 94% non‐Hispanic white). Biomarker and genetic data were analyzed to investigate sex differences and interactions in *TOMM40*‐G, indexed at SNP rs2075650, and levels of amyloid beta (Aβ42 and Aβ42/40), phosphorylated tau (ptau181 and ptau217), glial fibrillary acidic protein (GFAP), and neurofilament light chain (NfL) in *TOMM40*‐G carriers versus non‐carriers. models included interaction terms for sex and *APOE*4 with the *TOMM40*‐G allele, controlling for age and education.

**Result:**

*TOMM40*‐G carriers showed significant differences in plasma biomarker levels compared to non‐carriers; carriers displayed increased ptau217 (*p* = 1.16e‐5) and decreased Aβ42/40 ratios (*p* = 8.66e‐6) and Aβ42 (*p* = 0.013). Levels of Aβ42 (*p* = 3.2e‐9) and Aβ42/40 (*p* <2e‐16) were lower in APOE4 carriers compared to non‐carriers while ptau181 (*p* = 8e‐05) and ptau217 (*p* = 5.29e‐10) were higher. Sex differences were significant for mean levels of ptau217 (*p* = 0.006), GFAP (*p* = 4.50e‐12) and Aβ42 (*p* = 0.069). Overall, men exhibited higher ptau217 while women had higher Aβ42 and GFAP levels. Results indicated significant interactions between sex and *TOMM40*‐G for NfL (*p* = 0.026) and Aβ42 (*p* = 0.079), such that *TOMM40*‐G is associated with lower levels of Aβ42 in female carriers and higher levels of NfL in male carriers. Sex and *APOE*4 interactions were significant for Aβ42 (*p* = 0.025) and Aβ42/40 (*p* = 0.004) in which female carriers had lower Aβ42 and Aβ42/40 levels compared to male carriers.

**Conclusion:**

The findings suggest that the *TOMM40‐*G risk allele is linked to altered plasma biomarkers. Although further research is needed to clarify sex differences, our results indicate that *APOE*4 and the *TOMM40* rs2075650‐G may respectively interact with sex to affect AD biomarkers, underscoring complex sex‐specific AD risk.

